# Predictive Value of Nasal Nitric Oxide and Serum NOS2 Levels in the Efficacy of Subcutaneous Immunotherapy in Pediatric Patients with Allergic Rhinitis

**DOI:** 10.1155/2022/1679536

**Published:** 2022-08-16

**Authors:** Sihui Wen, Shenghao Cheng, Shaobing Xie, Hua Zhang, Junyi Zhang, Fengjun Wang, Shumin Xie, Zhihai Xie, Weihong Jiang

**Affiliations:** ^1^Department of Otolaryngology Head and Neck Surgery, Xiangya Hospital of Central South University, Changsha, Hunan, China 410008; ^2^Hunan Province Key Laboratory of Otolaryngology Critical Diseases, Changsha, Hunan, China 410008; ^3^National Clinical Research Center for Geriatric Disorders, Changsha, Hunan, China 410008

## Abstract

**Background:**

Subcutaneous immunotherapy (SCIT) is an effective therapy for allergic rhinitis (AR), but some AR patients still do not benefit from it. Nasal nitric oxide (nNO) and inducible nitric oxide synthase (iNOS/NOS2) act important roles in AR. This study aims to explore the abilities of serum NOS2 and nNO in predicting the clinical efficacy of SCIT in AR patients.

**Methods:**

We recruited 40 healthy controls (HCs) and 120 AR patients in this study. Serum NOS2 and nNO levels were compared between the two groups. In the AR group, patients underwent and finished 1-year of SCIT, and divided into the effective and ineffective groups, and the relationships between serum NOS2 and nNO levels and efficacy of SCIT were evaluated.

**Results:**

The serum NOS2 and nNO levels were higher in AR patients than HCs. In the effective group, the serum NOS2 and nNO levels were increased than the ineffective group. ROC curves presented that a combination of serum NOS2 and nNO exhibited promising predictive ability in predicting the clinical efficacy of SCIT.

**Conclusions:**

Serum NOS2 and nNO levels were enhanced in AR patients and might affect the efficacy of SCIT. The combined use of serum NOS2 and nNO levels could be a reliable and useful method for predicting the clinical efficacy of SCIT.

## 1. Introduction

Allergic rhinitis (AR) is one of the most common chronic inflammatory diseases of the upper airways, which is characterized by Th2 type inflammatory disease mediated by immunoglobulin E (IgE) [1]. Recent epidemiological studies reported that the prevalence of AR in Chinese adults increased by 6.5% in the last 6 years, and the number of patients with AR continued to increase [2, 3]. Although it is not life-threatening, the classical symptoms of AR, such as nasal itching, rhinorrhea, sneezing, and nasal obstruction, exhibit negative impacts on the patient's quality of life and work efficiency [4–6]. At present, the treatment of AR mainly includes environmental control, medications, allergen-specific immunotherapy (AIT), and surgery. Among them, AIT has been shown to be an effective treatment for IgE-mediated diseases, which is also considered to be the only treatment that can alter the natural course of AR [7, 8]. AIT can be administered either subcutaneously (SCIT) or sublingually (SLIT), and more and more evidences showed that SCIT was superior to SLIT in controlling allergic symptoms and decreasing medication consumption [9–11]. However, not all patients respond to SCIT, and the overall effectiveness rate is 52.0%-86.4% [3]. Currently, there is no available biomarker or method to predict the response to SCIT treatment. Therefore, it is necessary to find objective indicators or biomarkers which can be utilized to predict the response to SCIT in AR patients.

Inducible nitric oxide synthase (iNOS/NOS2) catalyzes the production of nitric oxide (NO) and plays an essential role in metabolic and inflammatory processes [12, 13]. Studies have shown that NOS2 was widely expressed in human bronchial epithelial cells, macrophages, endothelial cells, and vascular smooth muscle cells [14], and abnormal expression of NOS2 was involved in a variety of inflammatory diseases, including asthma, psoriasis, AR, and chronic rhinosinusitis (CRS) [12, 15, 16]. Allergic airway inflammation can promote the hyperactivity of NOS2 and then aggravate the production of NO concentration in the airway [17]. Sakthivel and Guruvayoorappan [18] found that serum NOS2 levels were elevated in murine models of carrageenan- and formaldehyde-induced inflammation, and the concentration was significantly reduced in murine treated with extracts with anti-inflammatory effects. NO acts as a signaling molecule involved in the regulation of various physiological functions of the body, including inflammation [19–21]. Previous studies suggested that NO could promote Th2-type inflammatory responses and eosinophilic inflammation, which contributed to the pathomechanism of allergic diseases [22]. Measuring the concentration of exhaled NO is considered as a noninvasive method to identify and monitor eosinophilic airway inflammation in airway inflammatory disease [23]. Prior publications demonstrated that nasal nitric oxide (nNO) and fractional exhaled nitric oxide (FeNO) levels were associated with upper and lower airway inflammation, respectively [24, 25]. However, few studies have explored the effect of nNO as an objective indicator to reflect the efficacy of AIT in AR patients. This study aims to explore the role of serum NOS2 and nNO levels in AR and evaluate their abilities in predicting the clinical efficacy of SCIT in AR patients.

## 2. Material and Methods

### 2.1. Participants and Settings

We recruited 120 AR patients and 40 age- and sex-matched healthy controls (HCs) from December 2019 to January 2020. All patients met the diagnostic criteria of AR referring to the allergic rhinitis and its impact on asthma (ARIA) guidelines [26]. The criteria for patient inclusion were listed as follows: (1) a history of allergic symptoms (nasal itching, rhinorrhea, sneezing, and nasal obstruction) for 2 years or more; (2) positive skin test results of Dermatophagoides farina (*Der f*) and/or Dermatophagoides pteronyssinus (*Der p*) (at least ++) and/or sIgE level for *Der f* or *Der p* >0.35 IU/ml; and (3) moderate-severe AR. Exclusion criteria for the patients include (1) age<18 years; (2) with other inflammatory diseases or autoimmune diseases; (3) with active asthma; (4) had a history of immunotherapy; (5) treated with antibiotics, corticosteroids, or antiallergic drugs within 4 weeks before the study; and (6) pregnant condition. Demographic and clinical information of the subjects were collected, and serum samples were collected before SCIT. In the AR group, 94 of 120 AR patients received standard SCIT for 3 years. All participants were asked to complete several questionnaires about their symptoms at baseline and during the whole schedule of SCIT.

### 2.2. Measurement of Serum NOS2

Five ml of fresh venous blood was collected from all subjects and stored at room temperature for 1 hour. All blood samples were centrifuged at 4°C (3000 rpm for 10 min), and supernatants were collected and stored in aliquots at -80°C for subsequent experiments.

Serum samples were thawed and centrifuged before use. Serum NOS2 levels were measured by the ELISA kit commercial (Multisciences, Hangzhou, China) according to the manufacturer's instructions. Briefly, the target antigen was immobilized by passive uptake on a 96-well polystyrene microtiter plate. A blocking buffer was added to saturate all unbound sites and then incubated with an antigen-specific unlabeled primary antibody. An antihuman enzyme-coupled secondary antibody was subsequently added to bind to the primary antibody. Generally, the secondary antibody is conjugated to horseradish peroxidase (HRP) and detected with an enhanced chemiluminescence substrate (ECL) [27].

### 2.3. Measurement of nNO

Participant held a filter with one nostril blocked, and another nostril was unblocked during the entire measurement. Every subject was requested to inhale air through the filter and then emit a whistle for at least 10 seconds without stopping. Before the examination, smoking and strenuous exercise were unallowed for at least one hour, and intranasal corticosteroids or oral antiallergy medication should be suspended for more than 3 days. The nNO levels were measured by the Nano Coulomb Breath Analyzer (Sunvou-CA2122).

### 2.4. Immunotherapy

SCIT was conducted as previously described [28]. All AR patients received Novo-Helisen Depot (NHD) allergen extracts (Allergopharma, Reinbek, Germany) from *Der f* and *Der p* at a 1 : 1 ratio. According to the manufacturer's instructions, SCIT consists of two phases: initial treatment phase and maintenance treatment phase. To achieve long-term efficacy, a treatment course of 3 years is recommended. The initial treatment phase started with the minimum dose of low concentration NHD NO.1 and gradually increased to the maximum dose of high concentration NHD NO.3. The injection interval was generally 7-14 days. For NO.1 and NO.2, the doses were increased from 0.1, 0.2, 0.4, to 0.8 ml. For NO.3, the dose was increased from 0.1, 0.2, 0.4, 0.6, 0.8, to 1.0 ml. In the maintenance treatment phase, patients were injected with 1.0 ml of NO.3 with an injection interval of 4 to 6 weeks. SCIT was conducted in the outpatient department under the guidance of allergy experts, and all patients were observed for >30 minutes before leaving. All adverse reactions were recorded throughout the whole treatment.

### 2.5. Clinical Efficacy Assessment

After 1-year follow-up, the symptoms and medication consumption were recorded throughout the course of treatment. The early clinical efficacy of SCIT was assessed based on the improvement of clinical symptoms and the reduction of medication consumption after 1 year of treatment. The symptoms of all AR patients were scored using the widely accepted total nasal symptom score (TNSS) and visual analogue score (VAS). The sum of medication consumption in the previous week was defined as the medication score (MS), which was recorded according to the recommendations of the World Allergy Organization: 1, 2, and 3 points for oral or intranasal antihistamines, intranasal glucocorticoids, and oral glucocorticoids, respectively [29]. The sum of total nasal symptom score and final MS was defined as nasal symptom and medication score (SMS). A reduction of SMS by at least 30% compared to baseline level was defined as effective SCIT; otherwise, the SCIT was considered to be ineffective [28].

### 2.6. Statistical Analysis

Numerical variables were expressed as mean ± standard deviation (SD), Student's *t*-test was used for normally distributed variables, and the Mann–Whitney *U* test was applied for nonnormally distributed variables. Categorical data were expressed as frequencies and percentages, and the difference was compared using the Chi-square test. Receiver operating characteristic (ROC) curves were performed to evaluate the potential value of serum NOS2 combined with nNO in predicting the clinical efficacy of SCIT. Bilateral *P* values below 0.05 were considered statistically significant. All statistical analyses were performed using SPSS 19.0 statistical software (IBM, Chicago, IL, USA).

## 3. Results

### 3.1. The Baseline Data for All Subjects

A total of 160 individuals were included in this study, including120 cases in the AR group, and 40 cases in the HC group. As shown in Table 1, then, NO levels, serum NOS2 concentrations and allergic comorbidities rates are significantly higher in the AR group than the HC group, and no statistical differences were observed in other clinical parameters, including gender, age BMI, and alcohol consumption between two groups. Among the AR group, a total of 94 patients received SCIT, and 26 patients preferred other treatment options.  Table 2 summarizes the characteristics of these 94 patients, including symptom scores and clinical variables.

### 3.2. Serum NOS2 and nNO Level in the AR Patients and their Relationships with Clinical Variables

**Figure 1:**
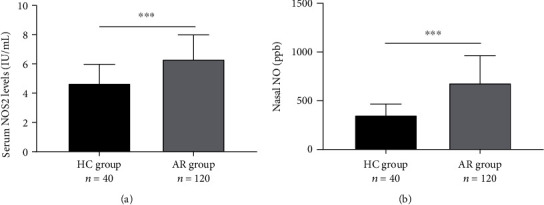
The levels of serum NOS2 and nNO in between the HC group and AR group. (a) Compared to the HC group, the serum levels of NOS2 were significantly increased in the AR group. (b) The nNO levels were notably higher in the AR group than HC group. NOS2: inducible nitric oxide synthase; nNO: nasal nitric oxide; HC: health control; AR: allergic rhinitis, ^∗∗∗^*P* < 0.0001.

**Figure 2:**
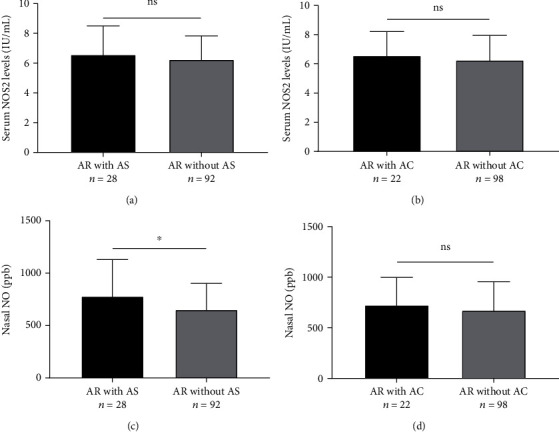
Expression levels of serum NOS2 and nNO in the AR subgroup. (a) Serum NOS2 levels were not different in AR with AS group and AR without AS group. (b) Similarly, serum concentrations of NOS2 were also not significantly different in the AR with AC group and AR without AC group. (c) nNO levels were significantly elevated in AR with AS group compared to the AR without AS group. (d) nNO levels were not significantly different in the AR with AC group and AR without AC group. NOS2: inducible nitric oxide synthase; nNO: nasal nitric oxide; AR: allergic rhinitis; AS: allergic asthma; AC: allergic conjunctivitis; ns: no significance, ^∗^*P* < 0.05.

Serum NOS2 and nNO levels were increased in the AR group compared to the HC group (*P* < 0.001, Figure 1). However, the serum concentrations of NOS2 were not significantly different in the AR subgroups (*P* > 0.05, Figures 2(a) and 2(b)). The nNO levels were significantly higher in the AR with AS subgroup than without AS subgroup (*P* < 0.05), but no statistically different in the AR with AC and without AC subgroups in Figures 2(c) and 2(d) (*P* > 0.05). To further explore the relationship between serum NOS2 and nNO levels and clinical variables, the correlation analysis was performed on the associations among age, BMI, disease duration, baseline VAS, and baseline TNSS. The results showed that serum NOS2 and nNO were inversely correlated with age and BMI, respectively, and serum NOS2 and nNO were positively correlated with each other (Table 3).

### 3.3. Variation of Serum NOS2 and nNO and the Connection with the Efficacy of SCIT

**Figure 3:**
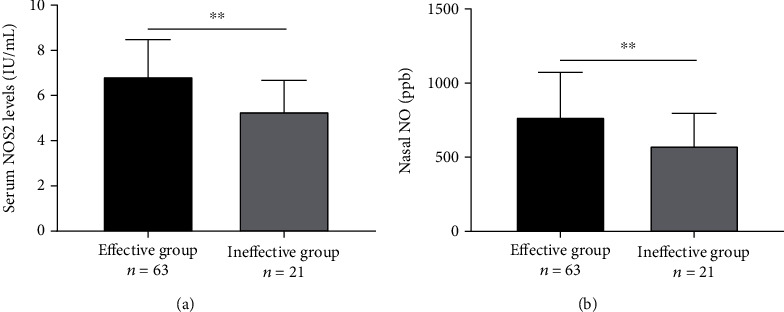
Serum NOS2 and nNO levels in between the effective group and the ineffective group. (a) Serum NOS2 concentrations were higher in the effective group than in the ineffective group. (b) nNO levels were clearly raised in the effective group in comparison with the ineffective group. NOS2: inducible nitric oxide synthase; nNO: nasal nitric oxide, ^∗∗^*P* < 0.01.

**Figure 4:**
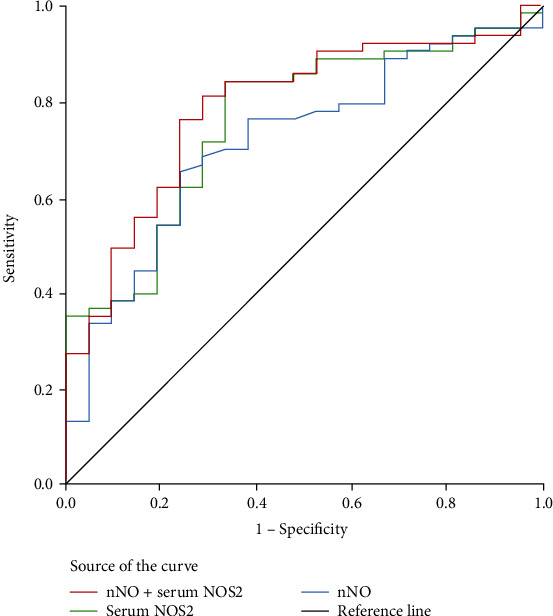
Receiver operating characteristic (ROC) curves for serum NOS2, nNO, and combined serum NOS2 and nNO distinguish responder from nonresponder in AR with SCIT. NOS2: inducible nitric oxide synthase; nNO: nasal nitric oxide; SCIT: subcutaneous immunotherapy.

Depending on the patients' response to SCIT, the AR group was further divided into the effective and the ineffective groups (Table 4). The results showed that the levels of serum NOS2 and nNO were distinctly upregulated in the effective group in comparison with the ineffective group (Figures 3(a) and 3(b)). For further investigation of the predictive value of serum NOS2 and nNO for the early efficacy of SCIT, ROC curves were performed. The ROC curve analysis revealed that the areas under the curve (AUC) were 0.759 (95%CI = 0.645-0.873) and 0.716 (95%CI = 0.596-0.837) of serum NOS2 and nNO, respectively. Interestingly, the AUC value was increased to 0.787 when combining serum NOS2 and nNO in predicting the efficacy (Figure 4). The detailed data are listed in Table 5.

## 4. Discussion

In the present study, our results indicated that levels of nNO and serum NOS2 were significantly higher in AR patients in comparison with the HCs, and their levels were clearly increased in the effective group than the ineffective group who were treated with SCIT. The ROC curve analysis revealed that the combination of these two indicators exhibited better predictive abilities than each single indicator. These results showed that serum NOS2 and nNO might affect the efficacy of SCIT, and the combined use of them might serve as a reliable and useful method for predicting the clinical outcome of SCIT in AR patients.

NOS2 was reported to be expressed in the nasal epithelial cell and associated with the production of NO, which contributed to aggravating eosinophilic inflammation [30]. Previous studies showed that elevated NOS2 expressions could enhance the release of NO, which was closely involved in vasodilation, microvascular leakage, smooth muscle relaxation, and abnormal glandular secretion [31]. As a common upper airway inflammatory disease, AR is typically characterized by a predominant Th2 response and eosinophil infiltration in nasal mucosa [32–34]. NO was proven to be pivotal in modulating the production of Th2 cytokines which were responsible for the migration of eosinophils into the airway mucosa [35]. Prior publications demonstrated that nNO could be used as a reproducible and noninvasive biomarker in respiratory diseases [36, 37]. Lv et al.'s study discovered that the measurement of nNO was useful for the early diagnosis of eosinophilic CRS with nasal polyps [38]. Our study indicated that the levels of both nNO and serum NOS2 were significantly higher in AR patients in comparison with the HCs, which was consistent with previous studies [39]. Moreover, we also found that serum NOS2 and nNO levels were negatively correlated with age and BMI, respectively (*r* = −0.239, *P* = 0.020; *r* = −0.214, *P* = 0.039). Accordingly, NOS2 was derived from airway epithelial cells which were gradually deteriorated with age [40]. Meanwhile, Holguin F et al. found that obesity was associated with lower plasma L-arginine/asymmetric dimethyl arginine (ADMA), which explained why exhaled NO was inversely related to BMI [41]. Given that, we suggested that NOS2 and nNO played crucial roles in the pathophysiology of AR, and age and BMI might be potential factors associated with the NOS2 and nNO levels.

SCIT was demonstrated to be an effective treatment for AR, but a certain proportion of patients still responds poorly to this therapy [42, 43]. The main aims of SCIT were to establish peripheral immune tolerance to allergens, suppress allergic responses, the balance of Th1/Th2 response, and induce IgG4 production [44]. Recent studies have found that serum-specific IgE/total IgE ratio and serum-specific IgE at baseline were considered to be potential predictors for treatment outcome [45, 46]. Parisi et al. [23] observed that nNO might serve as a predictive indicator of short-term SCIT efficacy in children with AR. In addition, Lee and his colleagues indicated that nNO could be a long-term biomarker for monitoring and prognostic of mucosal health in CRS [47]. However, the application of these biomarkers in clinical practice remains controversial because of discordant sensitivity and specificity. In this study, our results demonstrated that serum NOS2 and nNO levels were significantly elevated in the effective group than the ineffective group. Besides, ROC curves analysis showed that serum NOS2 and nNO exhibited potential abilities in predicting SCIT efficacy, and combined these two indicators showed significantly greater predictive value. Macrophages, as innate immune cells, are involved in the maintenance of allergen tolerance [48]. Previous studies confirmed that the production of IgG4 was triggered in vitro by M2b-like suppressor macrophages after AIT, and these macrophages could induce the production of IL-10, which suppressed the Th2 cell responses [48, 49]. Furthermore, macrophage subtypes can be interconverted during the process of reprogramming inflammatory environments and developing immune tolerance. A prior publication revealed that M1 macrophage polarization was observed in the early stage after viral infection and then gradually transformed to M2b macrophage polarization [50]. Taken together, we speculated that M1 macrophages were gradually transformed into M2b macrophages during SCIT, which were involved in the therapeutic mechanisms of SCIT and contributed to immune tolerance. On the other hand, nNO expression levels could reflect Th2 cytokine-induced type 2 inflammation and the number of eosinophils in the airways [51]. SCIT was identified to be effective by suppressing eosinophilic inflammation and promoting a deviation of Th2-type inflammatory response toward Th1. Therefore, nNO as an indicator associated with eosinophilic inflammation might reflect the efficacy of SCIT [51, 52]. Given that, we can infer that NOS2, an M1 macrophage marker, its concentrations, and nNO levels before SCIT might show synergistic effects and exhibit greater predictive values for the efficacy of SCIT. But the underlying mechanisms need to be further investigated.

The present study has several limitations. Firstly, the follow-up period was only 1 year, and the therapeutic evaluation might be biased and not representative. Secondly, the sample size was relatively small, and the participants were recruited from a single medical center, which might increase the risk of selection bias. Therefore, further investigation with a larger population and longer follow-up time was necessary to confirm the results of this study.

In conclusion, this study had certain strengths in terms of novel design. Our results proved that serum NOS2 and nNO were not only involved in the pathological process of AR but also affected the efficacy of SCIT. Combined use of serum NOS2 and nNO levels seems to be a reliable and useful method for predicting the clinical efficacy of SCIT in AR patients.

## Figures and Tables

**Table 1:** Demographics and clinical characteristics of patients between two groups.

Variables	AR group (*n* = 120)	HC group (*n* = 40)	*P* value
Sex			0.647
Male	55 (45.8%)	20 (50.0%)	
Female	65 (54.2%)	20 (50.0%)	
Age, years	31.7 ± 10.6	30.4 ± 7.6	0.480
BMI, kg/m^2^	22.9 ± 1.5	22.5 ± 1.3	0.215
Smoking	20 (16.7%)	8 (20.0%)	0.631
Alcohol consumption	10 (8.3%)	6 (15.0%)	0.362
nNO, ppb	684.2 ± 279.0	355.4 ± 109.8	*<0.001*
Serum NOS2(IU/ml)	4.7 ± 1.3	6.3 ± 1.7	*<0.001*
Disease duration, years	4.6 ± 2.6	—	—
Allergic comorbidities			
AS	28 (23.3%)	0 (0.0%)	*0.001*
AC	22 (18.3%)	0 (0.0%)	*0.004*
Baseline VAS	5.8 ± 3.5	—	—
Baseline TNSS	7.6 ± 2.8	—	—

BMI: body mass index; nNO: nasal nitric oxide; ppb: parts per billion; AS: allergic asthma; AC: allergic conjunctivitis; TNSS: total nasal symptom score; VAS: visual analogue scale.

**Table 2:** Baseline characteristics of the SCIT patients.

Variable	SCIT patients (*n* = 94)
Age: years	31.8 (10.8)
Sex: male/female	52/42
BMI, kg/m2	22.7 (1.4)
Disease duration, years	4.6 (2.6)
Smoking, yes/no	16/78
Alcohol consumption, yes/no	9/85
nNO, ppb	707.7 (287.1)
Serum NOS2(IU/ml)	6.6 (1.7)
Allergic comorbidities	
AS	19
AC	75
Baseline VAS	6.2 (2.2)
Nasal congestion	2.0 (0.8)
Runny nose	2.1 (0.8)
Sneeze	2.2 (0.7)
Itchy nose	1.8 (0.8)
Baseline TNSS	6.3 (1.7)

BMI: body mass index; nNO: nasal nitric oxide; ppb: parts per billion; AS: allergic asthma; AC: allergic conjunctivitis; TNSS: total nasal symptom score; VAS: visual analogue scale.

**Table 3:** Association between serum NOS2, nNO, and clinical variables in AR patients.

Variable	Serum NOS2 level		nNO	
	*r*	*P* value	*r*	*P* value
Age	-0.239	*0.020*	-0.137	0.189
BMI	-0.121	0.246	-0.214	*0.039*
Disease duration	0.140	0.180	-0.003	0.980
Baseline VAS	0.025	0.809	-0.003	0.980
Baseline TNSS	0.025	0.808	0.029	0.794
nNO	0.480	*<0.001*	—	—
Serum NOS2 level	—	—	0.480	*<0.001*

BMI: body mass index; nNO: nasal nitric oxide; TNSS: total nasal symptom score; VAS: visual analogue scale.

**Table 4:** Demographics and clinical characteristics of two groups.

Variables	Effective group (*n* = 63)	Ineffective group (*n* = 21)	*P* value
Sex			*0.016*
Male	23 (36.5%)	14 (66.7%)	
Female	40 (63.5%)	7 (33.3%)	
Age, years	31.1 ± 10.9	33.9 ± 10.9	0.327
BMI, kg/m^2^	22.7 ± 1.4	23.0 ± 1.2	0.356
Smoking	12 (19.0%)	2 (9.5%)	0.310
Alcohol consumption	9 (14.3%)	0 (0.0%)	0.067
nNO, ppb	773.2 ± 300.4	578.9 ± 216.9	*0.008*
Serum NOS2(IU/ml)	6.9 ± 1.6	5.3 ± 1.4	*<0.001*
Disease duration, years	4.7 ± 3.0	4.1 ± 1.4	0.342
Allergic comorbidities			
AS	14 (22.2%)	3 (14.3%)	0.443
AC	9 (14.3%)	4 (19.0%)	0.601
Baseline VAS	6.1 ± 2.2	6.0 ± 2.3	0.807
Baseline TNSS	8.2 ± 2.2	7.8 ± 2.4	0.506

BMI: body mass index; nNO: nasal nitric oxide; ppb: parts per billion; AS: allergic asthma; AC: allergic conjunctivitis; TNSS: total nasal symptom score; VAS: visual analogue scale.

**Table 5:** ROC results of different parameters for early predicting SCIT efficacy.

Variables	AUC (95% CI)	*P* value	Cut-off value	Sensitivity	Specificity
Serum NOS2 (IU/ml)	0.759 (0.645-0.873)	*<0.001*	5.5	84.1%	66.7%
Nasal NO (ppb)	0.716 (0.596-0.837)	*0.003*	681.0	65.1%	76.2%
Combined	0.787	*<0.001*	—	—	—

ROC: receiver operating characteristics; SCIT: subcutaneous immunotherapy; AUC: area under the curve; CI: confidence interval; ppb: parts per billion.

## Data Availability

The raw data supporting the conclusions of this article will be made available by the authors, without undue reservation.

## Abbreviations

AR: Allergic rhinitis
Th: T cell helper
IgE: Immunoglobulin E
AIT: Allergen-specific immunotherapy
SCIT: Subcutaneously immunotherapy
SLIT: Sublingually immunotherapy
iNOS/NOS2: Inducible nitric oxide synthase
NO: Nitric oxide
CRS: Chronic rhinosinusitis
nNO: Nasal nitric oxide
FeNO: Fractional exhaled nitric oxide
HCs: Healthy controls
ARIA: Allergic rhinitis and its impact on asthma
Der f: Dermatophagoides farina
Der p: Dermatophagoides pteronyssinus
ELISA: Enzyme-linked immunosorbent assay
HRP: Horseradish peroxidase
ECL: Enhanced chemiluminescence substrate
NHD: Novo-Helisen depot
TNSS: Total nasal symptom score
VAS: Visual analogue score
MS: Medication score
SMS: Symptom and medication score
SD: Standard deviation
ROC: Receiver operating characteristic
BMI: Body mass index
AS: Allergic asthma
AC: Allergic conjunctivitis
AUC: Area under the curve
CI: Confidence interval
IgG4: Immunoglobulin G4.
